# A simple but efficient tumor-targeted nanoparticle delivery system constructed by oleic acid

**DOI:** 10.1080/10717544.2022.2105447

**Published:** 2022-07-31

**Authors:** Jingxin Fu, Yian Wang, Haowen Li, Likang Lu, Meihua Han, Yifei Guo, Xiangtao Wang

**Affiliations:** Institute of Medicinal Plant Development, Chinese Academy of Medical Sciences & Peking Union Medical College, Beijing, China

**Keywords:** Oleic acid, elastic, nanoparticles, tumor-targeted

## Abstract

Oleic acid (OA) is a kind of monounsaturated omega-3 fatty acid that abounds in plants and animals which can induce apoptosis and has broad-spectrum inhibitory activity against a variety of tumor cell lines. However, OA is quite insoluble and thus inconvenient to be efficiently delivered in vivo. In this work, OA was fabricated into nanoparticles to generate OA elastic nanoparticles (OA-ENPs) with a particle size of 185.6 nm and good stability in various physiological media. OA-ENPs alone achieved a high tumor inhibition rate of 60.3% without significant side effect. More surprisingly, the resultant OA-ENPs displayed dose-dependent tumor targetability. Low dose of OA-ENPs (10 mg/kg) mainly distributed in the liver after intravenous injection, while high dose of OA-ENPs mainly distributed in tumor. At the high dose of 90 mg/kg, OA-ENPs accumulation in tumor reached nearly twice as that in the liver. Here we provide a simple but effective way to achieve excellent tumor targetability without the need of any surface modification of nanoparticles.

## Introduction

Nowadays, cancer remains one of the most devastating diseases in the world (Sung et al., 2021). The usage of nanoparticles achieves effective drug delivery using a mechanism known as the enhanced permeability and retention effect, which leads to improved anti-cancer efficacy and reduced side effect (Mitragotri, 2009). The initial success has been achieved for Doxil (Caelyx in Europe; PEGylated liposomal doxorubicin) and Abraxane (albumin-based paclitaxel) (Lammers et al., 2012). Compared with traditional rigid nanoparticles, elastic nanoparticles have a series of advantages in drug delivery: (1) prolonged circulation time. Elasticity can reduce nanoparticle’s uptake by liver MPS uptake, prolong the blood circulation. High deformable nanoparticles are easily trapped in the fenestrations of spleen for a short time, and then released back to the blood circulation (Liang et al., 2019). (2) Tumor accumulation. Soft nanomedicines outperform their stiff counterparts in tumor accumulation, partly due to prolonged blood circulation, partly due to their enhanced ability to pass across the blood vessels (Li et al., 2020; Zhang et al., 2021). (3) Tumor penetration. Most solid tumors are characteristics of tortuous blood vasculature, high interstitial fluid pressure, and a dense extracellular matrix. Soft particles are easier to squeeze and penetrate deeply into tumor parenchyma than rigid nanomedicines due to their deformability (Li et al., 2020).

Liquid pharmaceutical adjuvants could be fabricated into elastic nanoparticles due to their fluidity and deformability. Unsaturated fatty acids are usually in the form of liquid, they are beneficial nutrients and dietary supplements for human health (Burr & Burr, 1973; Zicha et al., 1999; Denyer, 2002). They also play essential roles in the biological activities of cells as major components of cellular membranes (Alessenko & Burlakova, 2002; Denyer, 2002). Tumor cells usually display an upregulated demand for unsaturated fatty acids to form various cellular membranes due to their vigorous cell division and proliferation.

Oleic acid (OA), as the prime unsaturated mono-fatty acid, could regulate cell membrane lipid structure to achieve anti-tumor effects (Yang et al., 2005). OA-chemotherapeutics conjugations displayed good in vivo anti-tumor efficacy (Luo et al., 2016). Furthermore, OA itself displays antitumor effects. It might influence the complex stages of the process of carcinogenesis, for example, the oxidative stress, alteration of the hormonal status, modulation of cell signaling transduction pathways, regulation of gene expression, and influence on the immune system (Solanas et al., 2002; Escrich et al., 2011).

Based on the above consideration, OA was fabricated into nanoparticles using mPEG_2000_-DSPE as a stabilizer. It was anticipated that the OA nanoparticles were elastic or flexible in contrast to common nanoparticles such as nanosuspensions and the resultant oleic acid elastic nanoparticles (OA-ENPs) could significantly improve the in vivo antitumor efficacy of free OA. Surprisingly, it was found that OA-ENPs, without the need of any tumor-targeting modification, displayed extremely high tumor accumulation (nearly twice as that in the liver) after high dose of intravenous injection, and OA-ENPs alone achieved a tumor inhibition rate of 60.3%. This paper provided a simple but efficient way for tumor-targeted drug delivery.

## Material and methods

### Materials

Oleic acid was purchased from BioRuler company. mPEG_2000_-DSPE was supplied by Shanghai Toyong Biotech company (Shanghai, China). DiR iodide [1-1-dioctadecyl-3,3,3,3-tetramethlindotricarboc-yanine iodide] (DiR) was purchased from AAT Bio Quest, Inc. Doxorubicin hydrochloride (DOX) for injection was obtained from Shenzhen Mian Luck Pharmaceuticals Inc. (Shenzhen, China). 3-(4,5-dimethylthiazol-2-yl)-2,5-diphenyltetrazolium bromide (MTT) were purchased from Sigma Aldrich (St. Louis, MO, USA). Icaritin was supplied by Nanjing Dasfbio Co. Ltd. (Nanjing, China). ELISA plate reader was purchased from Biotek (Winooski, VT, USA).

### Cell line and animals

The 4T1 (breast carcinoma) cell line was supplied by the Cell Resource Center, Peking Union Medical College (Beijing, China). Cell was cultured in RPMI 1640 medium with 10% fetal bovine serum (Thermo Fisher Scientific), streptomycin (100 U/mL), and penicillin (100 U/mL) at 37 °C in 5% CO_2_ (Sanyo, Osaka, Japan). Female BALB/c mice (20 ± 2 g, 6–8 weeks old) were obtained from Charles River Laboratory Animal Technology Co. Ltd. (Beijing, China). Before the experiment, animals grew for a week at 25 °C under a light-air flow environment with a relative humidity of 70 ± 5%.

### Preparation of OA-ENPs

OA-ENPs were prepared by the bottom-up method. Briefly, 30 mg OA and mPEG_2000_-DSPE with different feeding ratios (1:1, 2:1, 3:1, 4:1, w/w) were co-dissolved in 5 mL ethanol. The ethanol solution was dropped slowly into 10 mL deionized water under continuous magnetic stirring (450 r/min), followed by vacuum rotary evaporation at 45 °C to remove the ethanol and transparent OA-ENPs with light opalescence is obtained.

DiR-labeled OA-ENPs were prepared as the procedure mentioned above, expect that DiR was added in the ethanol solution with the mass ratio of OA:DiR being 40:1.

### Dynamic light scattering measurement

The particle size distribution and zeta potential (ZP) of OA-ENPs were detected by dynamic light scattering (Zetasizer Nano ZS, Malvern Instruments, UK) at 25 °C, which integrated phase analysis light scattering (λ = 633 nm) and noninvasive backscatter optics (scattering angle θ = 173°). What’s more, each sample was measured three times, and all data was mean ± standard deviation (SD).

### Morphological observation

Morphological characterization of OA-ENPs was observed using a JEM-1400 transmission electron microscope (JEOL, Tokyo, Japan). One drop of OA-ENPs diluted nanosuspension was dropped on the 300-mesh copper mesh and naturally dried. The samples were then stained with 20 μL 2% w/v phosphotungstic acid dye for 2 min. Then the morphology was observed using a transmission electron microscope at an accelerating voltage of 120 kV.

### Stability in various physiological media

OA-ENPs were respectively mixed with 10% glucose solution and 1.8% NaCl (1:1, v/v) and phosphate buffer (PBS, pH 7.4), simulated gastric fluid (1% pepsin in 1 mol/L diluted HCl), simulated intestinal fluid (1% pancreatin in pH 6.8 PBS, 0.01 M), and plasma (1:4, v/v) for incubation at 37 °C. The samples were taken out at different time intervals for particle size distribution determination by Dynamic light scattering (DLS) and each sample was performed in triplicate.

### Storage stability

OA-ENPs were stored at room temperature for 120 days. The particle size and Polymer dispersity index(PDI) value of the samples were measured by Zetasizer Nano ZS at 1, 2, 4, 8, 16, 30, 60, and 120 days. Each sample was performed in triplicate.

### Elasticity evaluation

Preparation of nonelastic nanoparticles (HICT-NPS): 15 mg of hydrous icaritin (HICT) and 5 mg of mPEG_2000_-DSPE with a mass ratio of 3:1 were co-dissolved in 5 mL mixed organic solvent (acetone/ethanol, 2:1, v/v), then dropped into 5 mL deionized water under continuous magnetic stirring (450 r/min), followed by vacuum rotary evaporation at 45 °C to remove the organic solvents, finally homogenized (1500 bar, 15 cycles) to obtain HICT nanosuspensions, which was concentrated by vacuum rotary evaporation at 60 °C to about 3 mg/mL corresponding HICT for use. The resultant HICT nanoparticles (HICT-NPs) was employed here as a rigid nanoparticles control for OA-ENPs.

**Figure 1.**
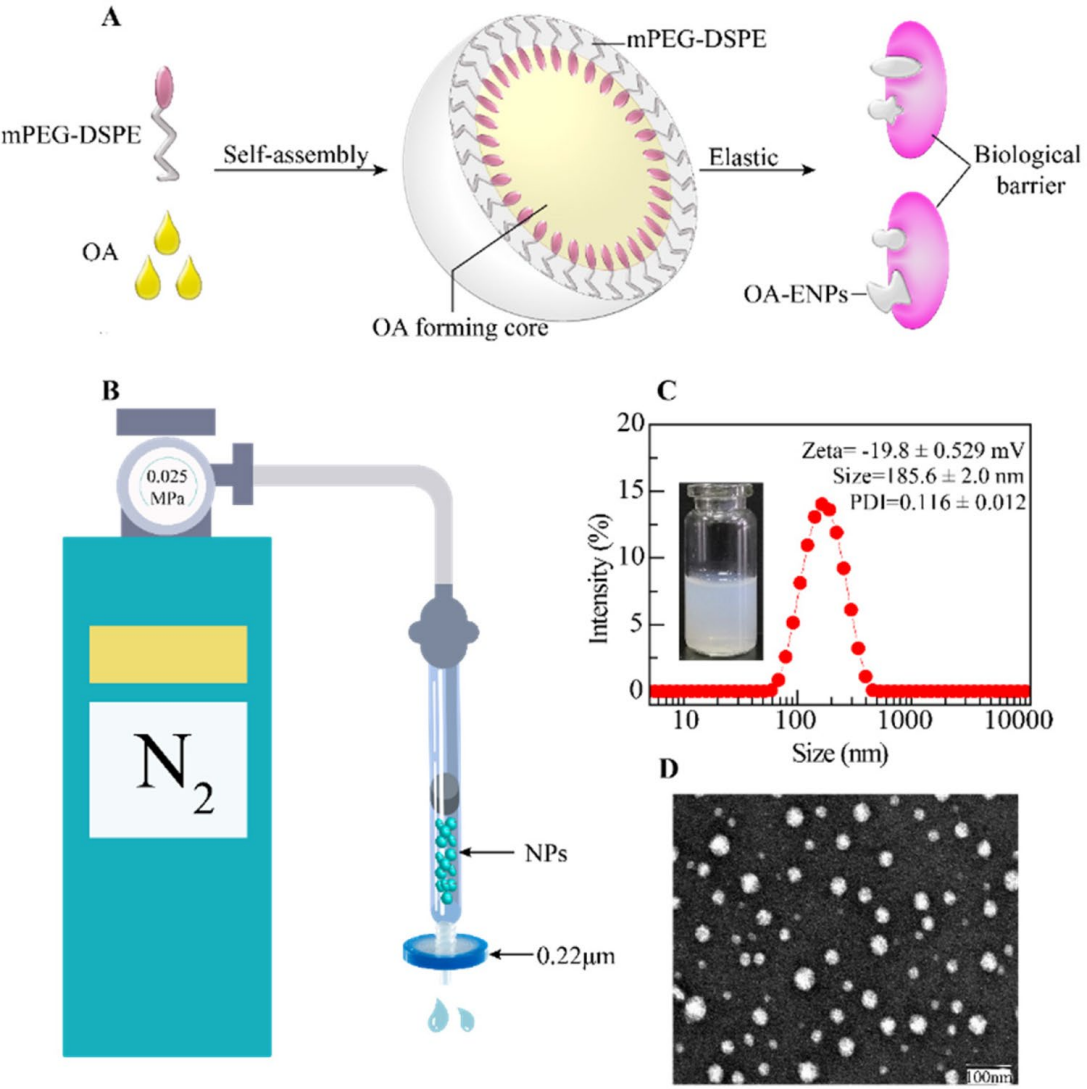
Structure of OA-ENPs and characterization of OA-ENPs. (A) Schematic diagram of OA-ENPs structure design and high elastic and plastic properties. (B) OA-ENPs denaturation ability verification device (the nitrogen pressure was 0.025MPa and the filter membrane aperture was 0.22 μm). (C) Apparent and particle size distribution of OA-ENPs. (D) TEM image of OA-ENPs (scale bar was 100 nm). Data was represented as the mean ± SD three times.

As shown in Figure 1, the elasticity of OA-ENPs was measured by their deformability during passing through small pores. OA-ENPs and HICT-NPs were forced by a continuous nitrogen flow of 0.025 MPa to pass through a 0.22 μm microporous filter membrane. The volume of nanoparticles or liposomes passed through the membrane within a fixed time period was accurately measured. Triple times of experiments were performed.

### Nanoparticle tracking analysis (NTA)

The number of nanoparticles in OA-ENPs was determined by Nanosight300 (NS300, Malvern Instruments, UK) at 25 °C, the instrument used a laser light source to illuminate the nanosuspension and an all-black background to provide signals to observe the Brownian motion of the particles with scattered light. NTA software could directly observe each nanoparticle and automatically track it, measure the particle size, and obtain the particles’ distribution in the whole system. The NTA Version is NTA 3.4 Build 3.4.003. Each sample was measured three times and the format of all data was mean ± SD.

### In vitro cytotoxicity

In vitro cytotoxicity evaluation of OA-ENPs was performed using the MTT assay. 4T1 murine breast cancer cells in the logarithmic growth phase were cultured in 96-well cell culture plates at 8000 cells per well and then cultured at 37 °C, 5%CO_2_ for 24 hours. The OA-ENPs and free OA solution (dissolve OA in DMSO) were diluted to different concentrations with RPMI-1640 medium and then added to a 96-well cell culture plate. After incubation for 48 hours in a cell culture incubator, 20 μL of MTT solution (5 mg/mL) was added to each well. The upper layer solution was discarded after 4 h. Lastly, 150 μL of DMSO solution was added to each well to fully dissolve the precipitate, and the value of absorbance was measured at a wavelength of 570 nm using an ELISA plate reader (Biotek, Winooski, VT, USA). The cell inhibition rate is calculated by the following formula:

$$\text{Inhibition rate}(\%)=(1-\frac{{\mathrm{OD}}_{e}}{{\mathrm{OD}}_{C}})\times 100\%$$

in which OD_e_ was the mean optical density of the experimental group and OD_c_ was the mean optical density of the blank control group. The IC_50_ was calculated by GraphPad Prism version 6.01 (GraphPad Software, Inc., La Jolla, CA, USA) using the sigmoidal dose–response variable curve-fitting method.

### In vivo antitumor efficacy

In vivo anti-tumor evaluation of OA-ENPs was performed using a female BALB/c 4T1 breast cancer mice model. When the mice weighed about 20 ± 1 g, each mouse was administrated with 0.2 mL of 4T1 cell suspension (RPMI-1640 medium dispersion, 4.0 × 10^7^ cells/mL) on the lower right side of the right forelimb. When the tumor volume reached about 100 nm^3^, the mice were randomly divided into six groups, which were respectively set as a negative group (0.2 mL normal saline), and a positive group (0.2 mL of DOX, 3 mg/kg), three OA-ENPs intravenous injection groups (10, 30, and 90 mg/kg were injected into the tail vein every other day), and an OA-ENPs gavage experimental group (OA, 180 mg/kg per day). All the mice were weighed every other day and the width *a* and length *b* of the tumors were measured using a Vernier caliper. The tumor volume was calculated by the formula: *V* = (*a* × *b*^2^)/2. After 15 days of administration, the mice were sacrificed 16 hour after the last dose by depolarization, and tumors and the major organs were collected and weighed. The tumor inhibition rate (TIR) was calculated according to the following formula:

$$\mathrm{TIP}(\%)=(1-\frac{W_{e}}{W_{n}})\times 100\%$$

in which *W_e_* were the mean tumor weight of the experimental group and *W_n_* was the mean tumor weight of the negative control group.

Liver index (LI) was calculated according to the formula below:

$$\mathrm{LI}=\sum_{i=1}^{6}\frac{W_{\mathrm{Liver}}}{W_{mi}}÷6$$

in which *W_Liver_*was the liver weight of mice and *W_mi_*was the body weight of mice.

Splenic index (SI) was calculated according to the formula below:

$$\mathrm{SI}=\sum_{i=1}^{6}\frac{W_{\mathrm{Spleen}}}{W_{mi}}÷6$$

in which *W_Spleen_*was the spleen weight of mice and *W_mi_* was the body weight of mice.

### In vivo biodistribution and tumor targetability

In order to know the in vivo biodistribution and tumor-targeting of OA-ENPs, DiR-labeled OA-ENPs were prepared, with DiR/OA mass ratio being 1:40. The 4T1 tumor bearing mice were made according to the same procedure as in the in vivo antitumor efficacy study. When the tumor volume reached about 1000 mm^3^, 30 mice were divided into three groups and intravenously injected with DiR-labeled OA-ENPs at the dose of 10 mg/kg, 30 mg/kg, and 90 mg/kg corresponding OA respectively. And 16 hours post dose, five mice in each group were sacrificed by depolarization, and tumors and the major organs were collected and imaged using the IVIS Living Image software (version 4.4, Caliper Life Sciences, Hopkinton, MA, USA) to observe the fluorescence intensity. The remnant five mice in each group were whole-body imaged using IVIS Living Image^@^ 4.4 (Caliper Life Sciences, Hopkinton, MA) at a set time after administration till the 72nd hours.

### Data analysis

The IC_50_ was calculated by GraphPad Prism 5 (GraphPad Software, Inc., La Jolla, CA, USA), and the experimental data collected was mathematically analyzed using Sass software, and statistical analysis was performed using T-test and one-way analysis of variance, when *p* < .05, it was statistically different.

## Results and discussion

### Preparation and characterization of OA-ENPs

Different feeding ratios of OA and mPEG_2000_-DSPE were examined and the results were illustrated in Table 1. It was clear the feed ratio of 3:1 (OA:mPEG_2000_-DSPE, w/w) achieved small particle size and most narrow size distribution, thus this feeding ratio was chosen to prepare OA nanoparticles for the subsequent use in this paper. Figure 1A illustrated the possible structure of the resultant OA nanoparticles, composed of OA core surrounded with DSPE-mPEG shell. DLS showed that OA nanoparticle was 185.6 ± 2.0 nm in mean particle size with a narrow PDI of 0.116 ± 0.012 and a surface zeta potential of −19.8 ± 0.529 mV (Figure 1C). OA-ENPs were spherical particles observed by transmission electron microscope (TEM) (Figure 1D). The particle size displayed by TEM was less than 100 nm, significantly smaller than that measured by DLS, this is because TEM measures the dry diameter of nanoparticles, while DLS measures the hydrodynamic diameter with the outer hydrated polyethylene glycol (PEG) chain corona (Varenne et al., 2016).

**Table 1.** The particle size and PDI value of OA-ENPs were prepared at different OA/DSPE-mPEG_2000_ feeding ratios (SD: standard deviation).

Feeding ratio	Size (nm)	PDI
1:1	199.5 ± 1.2	0.247 ± 0.016
2:1	183.1 ± 2.3	0.221 ± 0.005
3:1	185.6 ± 2.0	0.116 ± 0.012
4:1	234.6 ± 1.65	0.460 ± 0.022

### Elasticity of OA-ENPs

Flexible and morphologically variable nanoparticles had better tumor cell uptake than rigid ones (Touitou et al., 2001; Elsayed et al., 2006). The device shown in Figure 1B was used to measure the elasticity of OA-ENPs, using HICT-NPs (∼200 nm) as a control. As shown in Table 3, in 30 seconds, under the fixed nitrogen pressure of 0.025 MPa, the volumes of OA-ENPs and HICT-NPs passing through the 0.22 μm filter membrane were 0.9 ± 0.1 mL and 0.2 ± 0.05 mL. The transmission efficiency of OA-ENPs was 4.5 folds than that of HICT-NPs, indicating the better flexibility of OA-ENPs than the traditional nonelastic nanoparticle with similar number of particles.

**Table 2.** Storage stability of OA-ENPs.

Time	Size	PDI
1 day	185.6 ± 2.0	0.116 ± 0.012
2 days	180.3 ± 1.4	0.106 ± 0.022
4 days	185.6 ± 0.9	0.169 ± 0.011
8 days	196.7 ± 5.0	0.105 ± 0.014
16 days	199.8 ± 6.5	0.196 ± 0.028
30 days	187.7 ± 3.2	0.193 ± 0.032
60 days	192.5 ± 4.5	0.183 ± 0.044
120 days	193.8 ± 2.4	0.204 ± 0.003

**Table 3.** The 220 nm filtration efficiency of OA ENPs and HICT-NPs (mean ± SD).

Nanoparticles	Size (nm)	Time (s)	Volume (mL)	Number of particles/mL
HICT-NPs (3 mg/mL of HICT)	202.2 ± 1.4	30s	0.2 ± 0.05	2.07 × 10^11^±3.89 × 10^9^
OA-ENPs (3 mg/mL of OA)	185.6 ± 2.0	30s	0.9 ± 0.1**	2.28 × 10^11^±4.31 × 10^9^

The results are presented as the mean ± SD, *n* = 3. ^**^p < .01 vs. HICT-NPs.

### Stability of OA-ENPs

**Figure 2.**
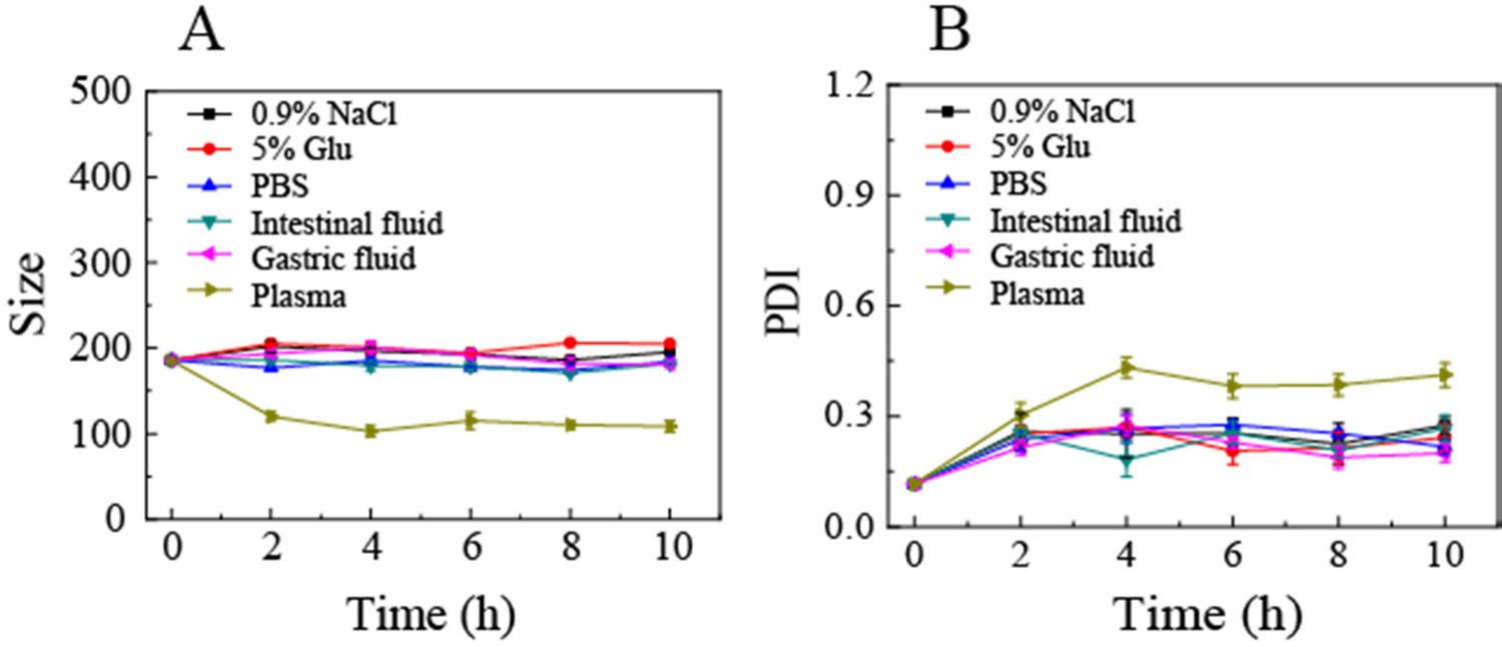
The average particle size (A) and PDI (B) of OA-ENPs in various physiological media.

As shown in Figure 2, OA-ENPs demonstrated stable particle size and PDI in various physiological media (1.8% NaCl, 10% Glu, PBS, simulated gastric fluid, simulated intestinal fluid, and plasma). The particle size maintained around 200 nm with a narrow PDI, suggesting that OA-ENPs suitable to various administration routes including oral administration and intravenous injection. OA-ENPs also demonstrated very good storage stability, with limited particle size and PDI value increase during the 120 days of storage at room temperature (Table 2) and no aggregation or sedimentation observed.

### Number of nanoparticles in OA-ENPs

The number of nanoparticles in 1 mg/mL, 3 mg/mL, and 9 mg/mL OA-ENPs were measured by NanoSight 300 to be 6.55 × 10^10^±3.60 × 10^9^ particles/mL, 2.28 × 10^11^ ± 4.31 × 10^9^ particles/mL, and 6.67 × 10^11^ ± 5.11 × 10^9^ particles/mL respectively, corresponding to about 0.0655 trillion/mL, 0.228 trillion/mL, and 0.667 trillion/mL. The determined nanoparticles number was nearly proportional to the concentration of OA-ENPs. The accurate determination of nanoparticles number in aqueous phase remains a challenge. Nanosight 300 is the only instrument in the market that can directly do so.

### In vitro antitumor activity

**Figure 3.**
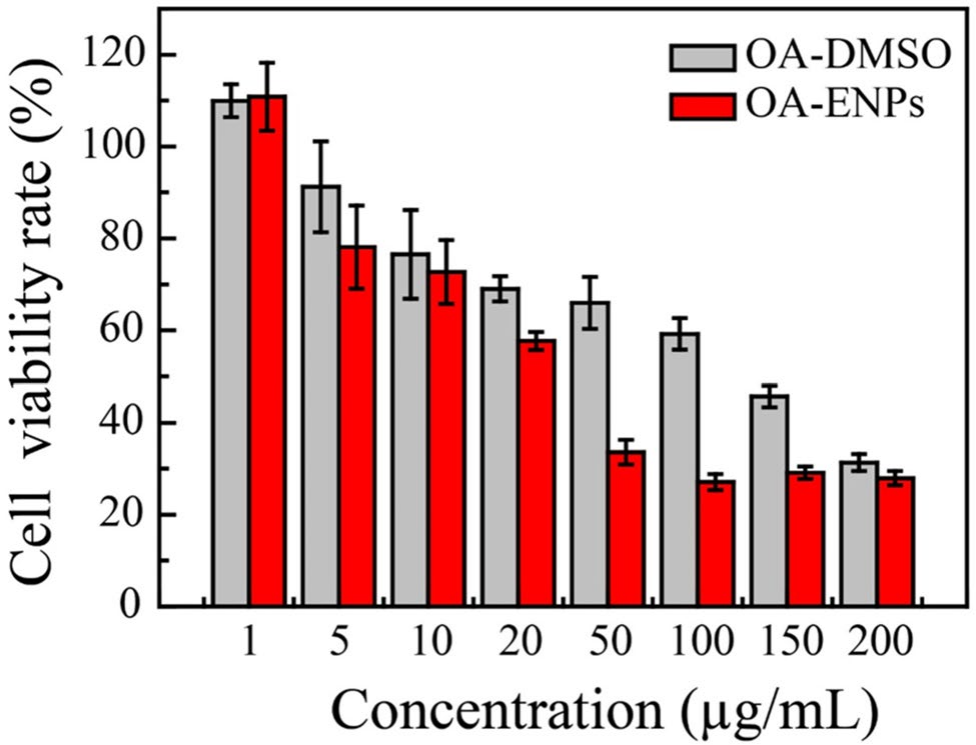
In vitro cytotoxicity of OA-ENPs and free OA solution against 4T1 cells after 48 hours incubation (mean ± SD).

The 4T1 murine breast cancer cell line was used to evaluate the anti-tumor effect of OA-ENPs in vitro. Different concentrations of OA-ENPs and OA-DMSO solution were co-cultured with 4T1 cells. As shown in Figure 3, OA-ENPs demonstrated higher tumor inhibition than free OA at every concentration. It was calculated that the IC_50_ value of free OA was 101.6 ± 1.4 µg/mL against 4T1 cell line, which was consistent with the reported data (Llor et al., 2003; Escrich et al., 2006). OA could induce apoptosis by regulating the membrane lipid structure of tumor cell membranes, but as an endogenous fatty acid, OA has a very low cytotoxicity. However, when OA was prepared into OA-ENPs, its IC_50_ value was decreased to 30.9 ± 4.8 µg/mL, probably due to the enhanced cellular drug uptake commonly led by nanoparticle encapsulation (Röhrig & Schulze, 2016).

### Anti-tumor efficacy of OA-ENPs in vivo

**Figure 4.**
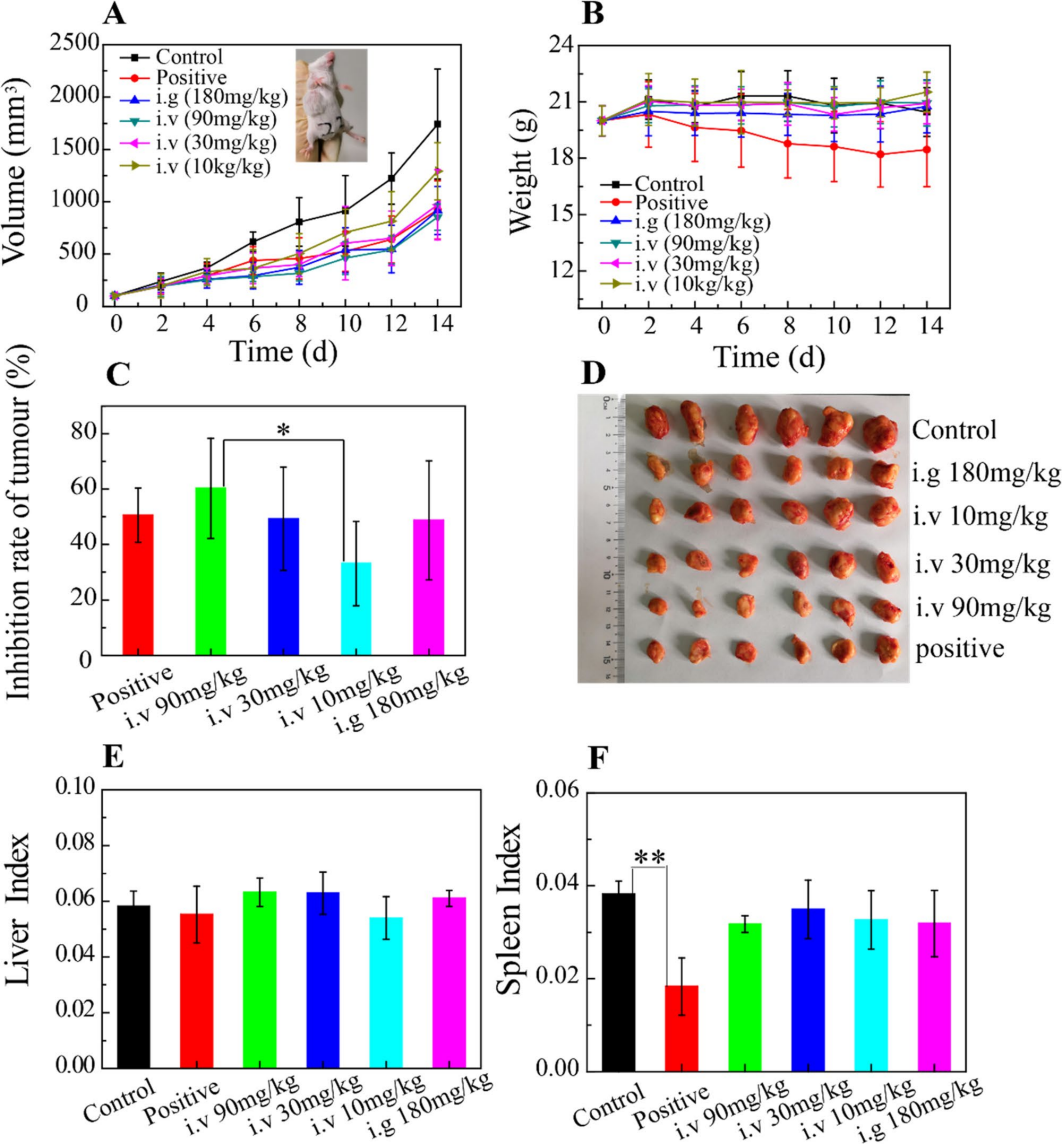
In vivo anti-tumor efficacy and in vivo safety of OA-ENPs against 4T1 tumor-bearing mice. (A) The tumor volume change curves. (B) Body weight change. (C) Tumor inhibition rate. (D) The features of anatomic. (E) Liver index. (F) Spleen index. For each animal, seven consecutive doses were given every other day. Data represent mean ± SD (*n* = 6). Abbreviations: i.v, intravenous; i.g, intragastric administration.

The anti-tumor efficacy of OA-ENPs was performed by 4T1 breast cancer mice model and the safety was evaluated by liver and spleen indexes. As shown in Figure 4A, the tumor volume of the negative group grew rapidly during the experimental period, while the growth of tumor was significantly inhibited in all OA-ENPs treated groups. Tumor inhibition rate of the positive group (DOX, 3 mg/kg, i.v.) reached 50%. As shown in Figure 4C and D, tumor inhibition rates of intravenously injected OA-ENPs group were 33.2%, 60.3%, and 49.3% respectively at the dose of 10 mg/kg, 30 mg/kg, and 90 mg/kg, showing good dose-dependence. The positive group receiving 3 mg/kg of DOX showed a TIR of 50%, similar to that of the middle dose of OA-ENPs group. In contrast, daily oral administration of OA-ENPs at 180 mg/kg only resulted in a TIR of 48.7%, which was comparable to the TIR of 1/6 dose (30 mg/kg) of intravenously administrated OA-ENPs. By the help of nanotechnology, OA itself achieved good antitumor efficacy.

During the whole process of the experiment, all the mice treated with OA-ENPs were in good condition. The mice treated with normal saline or DOX curled up, less active with unsmooth hair, especially those in DOX group. The body weight change profiles (Figure 4B) demonstrated that the mice in DOX group experienced a continuous decrease in body weight (from 20 g to 18 g in average), while the other group basically maintained their body weight, indicating that OA-ENPs had higher safety than DOX. This was in accordance with the liver index (Figure 4E) and spleen index (Figure 4F) data, for which DOX group showed significantly reduced spleen index in contrast to normal saline group and other groups (*p* < .01). As for liver index, no statistical difference was observed among all of the groups. The biological toxicity of DOX has been widely reported. Comparatively, OA-ENPs with better TIR and safety may be useful in the future tumor treatment, though more and further work need to be done.

### Biodistribution of OA-ENPs in vivo

**Figure 5.**
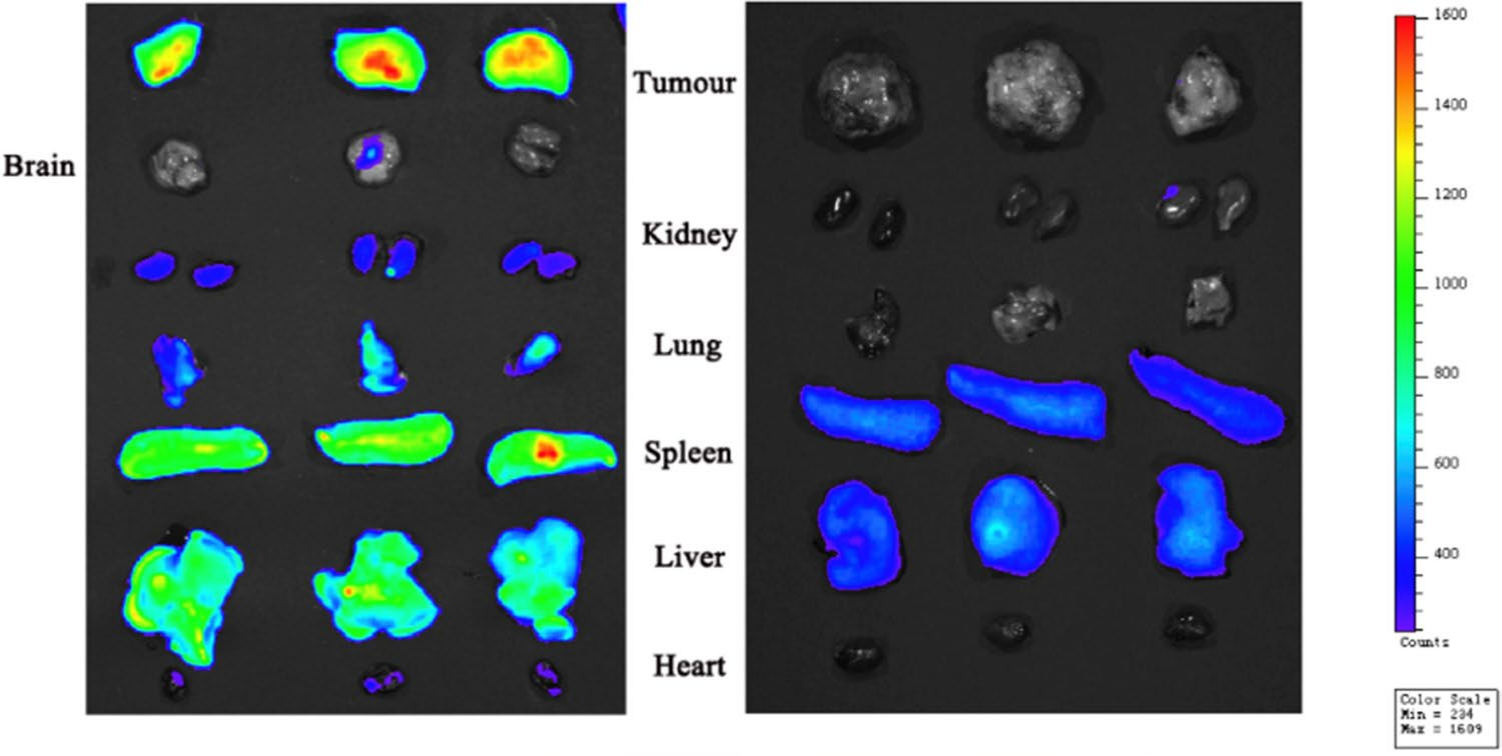
Tissue distribution of DiR-loaded OA-ENPs by intravenous (Left) and tissue distribution of DiR solution by intravenous (Right).

For the last dose in the in vivo antitumor efficacy study, three mice in the high dose OA-ENPs group were intravenously injected with DiR labeled OA-ENPs instead of OA-ENPs. It turned out that OA-ENPs showed much higher accumulation in tumor than in the liver (Figure 5). So, a systematic study was performed to verify the tumor-targetability of OA-ENPs and meanwhile to examine whether such good tumor-targetability is inherent to OA-ENPs themselves or variable along with dose.

**Figure 6.**
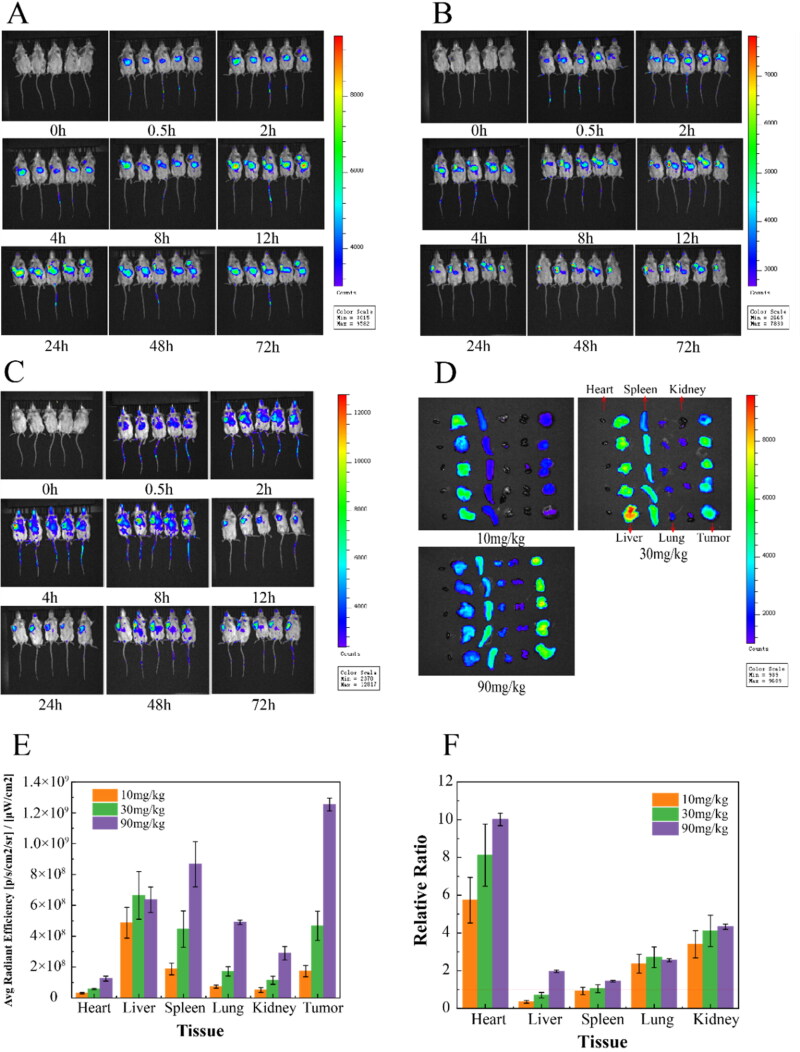
Dynamic distribution and tissue distribution of OA-ENPs in vivo. (A) Dynamic distribution of 10 mg/kg OA-ENPs in vivo. (B) Dynamic distribution of 30 mg/kg OA-ENPs in vivo. (C) Dynamic distribution of 90 mg/kg OA-ENPs in vivo. (D) Tissue distribution of OA-ENPs in vivo. (E) Fluorescence value per unit area of each tissue. (F) The ratio of fluorescence per unit area of the tumor to other tissues. Data represent mean ± SD (*n* = 5).

As shown in Figure 6A, at the low dose of 10 mg/kg, OA-ENPs were mainly distributed in the liver at first, their accumulation in tumor could be obviously observed only at the 8th hour, peaked at 12th to 24th. But it was evident that the liver held the strongest fluorescence throughout the observation period of 72 hours. In case of 30 mg/kg (Figure 6B), the liver also held the strongest fluorescence throughout the observation period, but the accumulation of OA-ENPs in tumor was much earlier this time. The 2nd hour witnessed the significant fluorescence in tumor, which was maintained throughout the whole observation period, overpassed the fluorescence intensity in the liver 24 hours later. Quite differently, in case of the 90 mg/kg (Figure 6C), the situation was reversed, tumor, instead of the liver, held the strongest fluorescence throughout the observation period. At each timepoints, OA-ENPs accumulation in tumor was obviously higher than that in the liver.

Direct imaging of the isolated tumor and organs could give more accurate information about OA-ENPs biodistribution. As shown in Figure 6D–F, at the dose of 10 mg/kg, OA-ENPs mainly accumulated in the liver with the fluorescence_tumor/liver_ being about 0.37. When the dose was increased to 30 mg/kg, OA-ENPs still preferred to distribute in the liver, while the fluorescent distribution in spleen and tumor was also elevated. At the dose of 90 mg/kg, OA-ENPs mainly accumulated in tumor, followed by that in the spleen and in the liver, with the fluorescence_ratio tumor/liver_ was increased to unbelievable 1.97, indicating the excellent tumor targetability. Now it was clear that the tumor targetability of OA-ENPs showed an obvious dose-dependence.

Ouyang et al. (2020) in their paper titled ‘*The dose threshold for nanoparticle tumor delivery’ *discovered a simple way by merely increasing the dose (actually the number of nanoparticles in a single dose) could effectively improve the tumor delivery and therapeutic efficacy of nanomedicine. They proved that a single dose above 1 trillion (1 × 10^12^) of nanoparticles in mice could overwhelm Kupffer cell’s uptake, nonlinearly decrease the liver clearance, and consequently increase the accumulation of nanoparticles in tumor (Ouyang et al., 2020). So, we examined that particle number of OA-ENPs using Nanosight 300 (NS300, Malvern Instruments, UK) and found that, at the dose of 90 mg/kg of corresponding OA, the number of nanoparticles injected for one mouse reached 0.133 trillion (0.667 trillion/mL, 0.2 mL injected). Similarly, the particle number for single dose was 0.0456 trillion/mouse for 30 mg/kg, 0.0131 trillion/mouse for 10 mg/kg.

We think the major reason that 90 mg/kg of OA-ENPs displayed much better tumor targetability than 30 mg/kg of OA-ENPs and 10 mg/kg of OA-ENPs was the result of dosing effect, which was later confirmed by Hui Ao et al. (2022).

But the over-threshold dosing effect may not be the only mechanism behind. As in Hui Ao’s work, the highest relative tumor targetability (fluorescence_tumor/liver_) through over-threshold dosing was only 1.345 using PEGylated liposomes. However, in our work, OA-ENPs at about 1/8-threshold dose achieved better relative tumor targetability with the fluorescence_tumor/liver_ being nearly 2.0. The elasticity of OA-ENPs and the much higher demand of tumor cells for unsaturated fatty acids than normal cells due to their vivid cell division and proliferation (Peck & Schulze, 2016; Röhrig & Schulze, 2016) may also made contribution. It was guessed from this study that the threshold dose for deformable nanoparticles such as OA-ENPs may be less than one trillion, meanwhile the flexibility may have synergistic action with dosing effect in tumor-targeted drug delivery.

It may not be an isolated event that nanoparticles made from unsaturated fatty acid help improve tumor targetability. Hui Ao et al. (2022) fabricated *Annona squamosa* seed oil (ASSO) into nanoparticles using TPGS as a stabilizer. ASSO was mainly composed of OA and linoleic acid, together with a small amount of stearic acid and palmitic acid, of which unsaturated fatty acids account for about 70%. The resultant ASSO-NPs showed similar particle size (about 194 nm) and high accumulation in tumor (fluoresence_tumor/liver_ =1.02). In addition, ASSO-NPs also significantly improved tumor accumulation of Annonaceous acetogenins, (ACGs) (a kind of effective fraction from *Annona squamosa*) in contrast to common nanoparticle (ACGs-NPs), with the fluorescence_tumor/liver_ increased from 0.88 to 1.29.

In general, a simple but efficient way on the basis of OA for tumor-targeting drug delivery was provided in this paper. Any antitumor agent, as long as being soluble in OA, theoretically can be benefited from this strategy.

## Conclusions

Although OA has demonstrated inhibitory activity against a number of tumor cell lines and OA conjugate of many chemotherapeutics showed significantly improved antitumor efficacy, so far, the relatively systematic in vivo antitumor study on OA itself has not been documented, probably due to the poor solubility of OA and the resultant inconvenience for its in vivo delivery. In this work, OA was prepared into nanoparticles using DEPE-mPEG_2000_ as a stabilizer, the obtained OA-ENPs were about 185.6 nm in mean diameter, showed good elastic property, good stability in various physiological media and on shelf. Nanotechnology enhanced the in vitro antitumor activity of free OA and OA-ENPs alone realized a high in vivo tumor inhibition rate of 60.3% in 4T1 bearing mice mode. More surprisingly, it was discovered that the dose increase could shift the main biodistribution of intravenously injected from liver to tumor, with main distribution in the liver at low dose, while main distribution in tumor at high dose. At the dose of 90 mg/kg, OA-ENPs accumulation in tumor reached nearly twice as that in the liver, which was seldom achieved in the field of nanomedicine. The over-threshold dosing effect was believed to be the major reason behind, meanwhile, the elasticity of OA-ENPs and the great demand of tumor cells for OA may also make contribution. All in all, this paper provided a simple but effective way to achieve excellent tumor targetability, which may benefit many agents related to tumor therapy as long as they show solubility in OA. It was guessed that the threshold dose for deformable nanoparticles such as OA-ENPs may less than one trillion, meanwhile the flexibility may have synergistic action with dosing effect in tumor-targeted drug delivery.
